# Hyperinfectivity: A Critical Element in the Ability of *V. cholerae* to Cause Epidemics?

**DOI:** 10.1371/journal.pmed.0030007

**Published:** 2005-12-06

**Authors:** David M Hartley, J. Glenn Morris, David L Smith

**Affiliations:** **1**Department of Epidemiology and Preventive Medicine, University of Maryland School of Medicine, Baltimore, Maryland, United States of America; **2**Fogarty International Center, National Institutes of Health, Bethesda, Maryland, United States of America; Imperial College LondonUnited Kingdom

## Abstract

**Background:**

Cholera is an ancient disease that continues to cause epidemic and pandemic disease despite ongoing efforts to limit its spread. Mathematical models provide one means of assessing the utility of various proposed interventions. However, cholera models that have been developed to date have had limitations, suggesting that there are basic elements of cholera transmission that we still do not understand.

**Methods and Findings:**

Recent laboratory findings suggest that passage of *Vibrio cholerae* O1 Inaba El Tor through the gastrointestinal tract results in a short-lived, hyperinfectious state of the organism that decays in a matter of hours into a state of lower infectiousness. Incorporation of this hyperinfectious state into our disease model provides a much better fit with the observed epidemic pattern of cholera. These findings help to substantiate the clinical relevance of laboratory observations regarding the hyperinfectious state, and underscore the critical importance of human-to-human versus environment-to-human transmission in the generation of epidemic and pandemic disease.

**Conclusions:**

To have maximal impact on limiting epidemic spread of cholera, interventions should be targeted toward minimizing risk of transmission of the short-lived, hyperinfectious form of toxigenic *Vibrio cholerae*. The possibility of comparable hyperinfectious states in other major epidemic diseases also needs to be evaluated and, as appropriate, incorporated into models of disease prevention.

## Abstract 2

Adjusting cholera models to take into account a short-lived hyperinfectious state proposed by recent experimental data improves their fit with real outbreak data.

## Introduction

While advances in medicine and public health have vanquished many pandemic diseases, 52 nations reported 142,311 cholera cases and 4,564 deaths in 2002, though these statistics are thought to represent a small subset of actual cases and deaths globally due to poor surveillance and under-reporting [1]. A complex web of interactions between the human host, pathogen, and environment are associated with the seasonal epidemics of cholera seen in endemic regions. The risk factors for cholera are varied and stem from multiple transmission pathways, including direct person-to-person contact and indirect transmission through the environment (e.g., food and water contamination) [2].

In epidemic situations, a fundamental question regarding the epidemiology of cholera is: what is the relative importance of human-to-human (i.e., fecal-oral) versus environment-to-human transmission (i.e., exposure to the environmental reservoir of *Vibrio cholerae*)? Answering this question may allow us to predict the onset, and potentially the intensity, of epidemics in endemic regions, as well as the speed and intensity of spread of cholera as it emerges in naïve regions. It may also afford us insight into new prevention, intervention, and control strategies to limit or prevent cholera transmission.

There are clues to the answer. Epidemic cholera is characteristically explosive in nature; when introduced into populations lacking prior immunity to the organism, spread through the population is measured in weeks, and involves all age groups [3,4]. Among more than 200 documented serogroups of *V. cholerae,* epidemic disease has been linked almost exclusively with serogroups O1 and O139. “Epidemic” strains colonize the small intestine and elaborate an enterotoxin (cholera toxin), which stimulates water and electrolyte secretion by intestinal endothelial cells and leads to massive fluid loss and profuse diarrhea. In volunteer studies, the frequency and severity of cholera has been correlated with inoculum [5,6]. The existence of such a dose-response relation (though typically difficult to measure) implies that the ability to infect a host is a key determinant of disease. Infective doses of environmental *V. cholerae* are thought to be in the range of 10^2^–10^3^ cells [2]. When individuals in a population are challenged with a dose many times larger than the ID_50_ (the infectious dose sufficient to produce frank disease in 50% of those exposed), the majority will be very likely to develop disease; an “explosive” epidemic will result. When challenged with a small fraction of the ID_50_, transmission will develop less rapidly, be less intense, and the outbreak will develop less rapidly. Thus, the inoculum of *V. cholerae* relative to the ID_50_ is key to understanding the intensity of cholera transmission.

In this context, recent experimental observations suggest that the *V. cholerae* ID_50_ depends upon the length of time the pathogen has existed outside the host. Passage of *V. cholerae* O1 Inaba El Tor through the human host appears to transiently increase the infectivity of *V. cholerae* [7]. Laboratory experiments demonstrate that when inoculated into the intestines of mice via gavage feeding, freshly shed *V. cholerae* greatly out-competes bacteria grown in vitro, by as much as 700-fold. The competitive advantage is short-lived; after standing 18 h, freshly shed *V. cholerae* organisms lose their competitive advantage. The advantage is maintained, however, for at least 5 h when incubated in pond water at room temperature. Comparing freshly shed vibrios to those not freshly shed, a different set of genes were up-regulated and these are thought to be responsible for faster bacterial growth in the gastrointestinal tract and increased shedding. Such observations suggest that passage of *V. cholerae* O1 Inaba El Tor through the human gastrointestinal tract results in a short-lived, hyperinfectious (HI) state. Recently, a second observation of a HI state in *V. cholerae* O1 El Tor has been published [8]. Whether similar states exist in other strains, biogroups, or serotypes of *V. cholerae* is unknown.

If indeed such a state exists, this hyperinfectivity is key to understanding the explosive nature of human-to-human transmission in outbreaks. Contact with freshly shed *V. cholerae* (lower ID_50_) is much more likely to cause disease than similar contact with *V. cholerae* shed much earlier (higher ID_50_). Moreover, since the decay from the HI state occurs within hours, it suggests that rapid local transmission through the direct route is responsible for rapid spread and the “explosive” nature of cholera epidemics, while the much slower environmental route accounts for much slower dynamics. Below we will use mathematical modeling to demonstrate that the existence of a transient HI state and the attendant reduction in ID_50_ explains the explosiveness of cholera epidemics. It is also consistent with other observed facets of cholera epidemiology and has concrete implications for public health strategies in prevention and intervention.

## Methods

Codeço has modeled the traditional picture of cholera, in which the pathogen has no transient HI state (Figure 1A) [9]. The model applies to a population of *N* people who are born and die on average at the rate *b*. Infections, *I,* are caused by ingesting water contaminated with *B* vibrios per ml. Ingestion occurs at the rate *β*. When *B* equals *κ*, the probability of ingestion resulting in disease is 0.5. Cases contribute to *V. cholerae* in the aquatic environment at a rate *ξ* and cases cease to be infectious at the rate *γ*. Vibrios shed into the aquatic environment lose viability at the rate δ.

**Figure 1:**
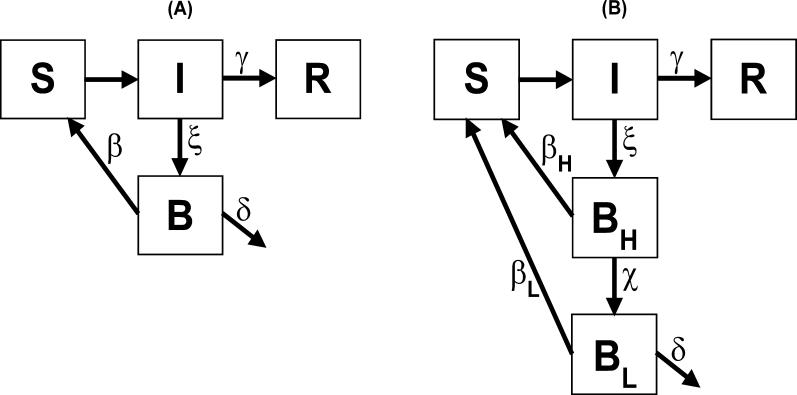
Models of Cholera Transmission (A) Model of cholera transmission, neglecting a HI state of *V. cholerae*. Simple model reflecting a picture of contagion in which susceptibles (S) become infectious (I) after consuming concentrations (shown by B) of *V. cholerae*. Individuals may achieve a transient immunity (R) after recovery. (B) Model of cholera transmission, incorporating a HI state of *V. cholerae*. This model includes contagion of *V. cholerae* that is HI (H) for a brief time after shedding and decays into a state of lower infectiousness (L). Greek letters denote rates of transitions between the different states, as described in Table 1.

**Table 1:**
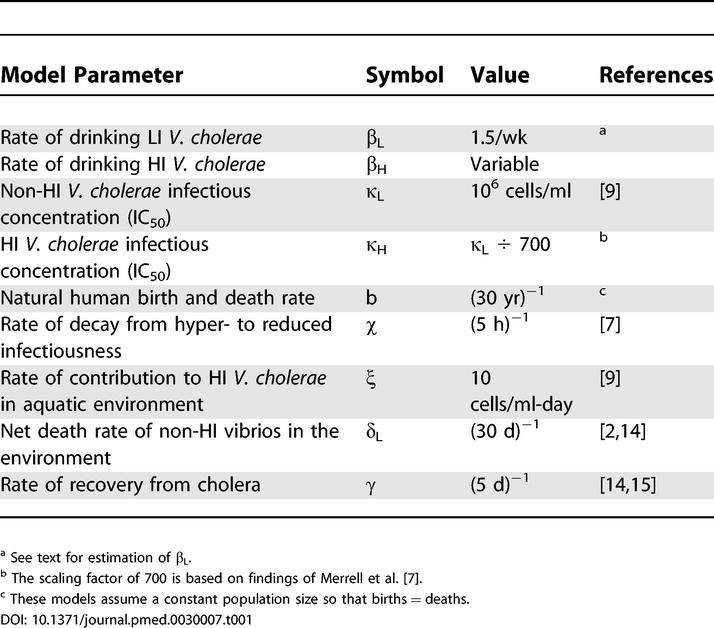
Model Parameters and Values

For any such model, the basic reproductive number, *R_0_,* provides a threshold for an epidemic to occur. Biologically speaking, *R_0_* is the number of cases per case at the beginning of the outbreak. If *R_0_* < 1, then a pathogen introduced into an immunologically naïve population will eventually die out. When *R_0_* > 1, endemicity is possible. At the same time, *R_0_* implicitly defines a timescale—the average time it takes for a pathogen to complete one generation. The larger *R_0_* and the shorter the generation, the more explosive epidemic transmission will be. The basic reproductive number for the model in [9] (no HI state) is the product of three epidemiologic factors: the total amount of *V. cholerae* shed into the environment (the term in the first pair of parentheses in the expression for *R_0_* below); the number of new cases per unit time generated by the shed pathogens (the term in the second pair of parentheses below); and the expected time that *V. cholerae* in the environment remains infectious before losing viability (the term in the third pair of parentheses below).



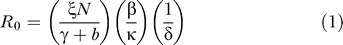



Estimation of the numerical value of *R_0_* for any disease is a difficult task. The above expression is useful in that it illustrates the quantities upon which the basic reproduction number depends, and their relative importance. Estimating the absolute value of *R_0_* from this expression, however, would entail knowledge of all variables in the expression, including some that are difficult to estimate directly. Many of these have been estimated in the literature (Table 1), but others are less well known and/or are highly variable. The parameter *β* (effectively, the population average rate of drinking potentially contaminated water) in particular is difficult to estimate. Codeço takes *β* = 1/day as a baseline, which yields *R_0_* = 15. Alternatively, *β* can be estimated algebraically by approximating *R_0_* roughly using the average age at first infection *(A)* and the mean lifetime of the population *(L)* in endemic areas.



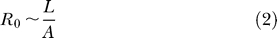



This method for estimating *R_0_* depends on many assumptions that may not be satisfied for cholera [10]. On the other hand, the L/A is a useful index of *R_0_* for cholera in endemic areas since both *L* and *A* are readily measured. In four epidemics where estimates of *L* and *A* were both available, *R_0_* values ranged from approximately 3 up to 15 (Table 2), with an average of ~ 8.7.

**Table 2:**
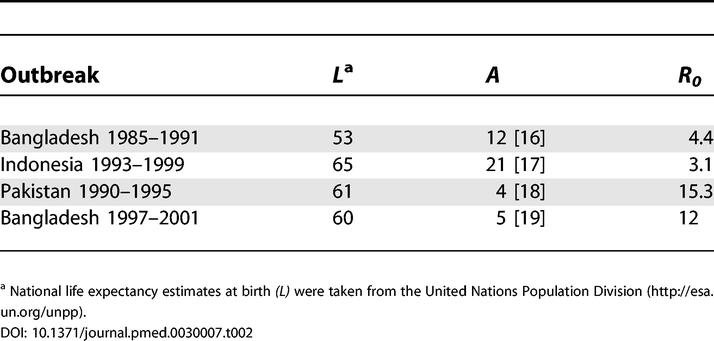
Estimates of *R_0_* from Past Cholera Epidemics

As described below, we have modified the model of cholera described in [9] to include a HI state of *V. cholerae*. The model is expressed in terms of differential equations and analyzed using standard methods [11,12]. The temporal epidemic and endemic behavior of cholera are estimated from the model through computer simulation of the model. The difference in the timing and intensity of epidemics that the HI state introduces relative to a single state of lower infectivity is illustrated by plotting simulated cholera cases through time when the HI state is both included in and excluded from the model. All simulations are carried out using the R (version 2.1.1, http://www.r-project.org/) *odesolve* library.

We have extended the Codeço model to incorporate a state of hyperinfectivity (Figure 1B). In this modified model, infections, *I,* are caused by ingesting water contaminated with *B_H_* HI vibrios per ml or *B_L_* non-HI vibrios per ml. Ingestion of HI vibrios occurs at the rate *β_H_* while ingestion of non-HI vibrios occurs at the rate *β_L_*. When *B_H_* equals *κ_H_*, the probability of ingestion resulting in disease is 0.5, and similarly for *B_L_* and *κ_L_*. In other words, the models assume that the relationship between infection rates and the density of cholera is described by a saturating function *β B/(B + κ)*. Vibrios in the HI state decay into a state of lower infectivity (“non-HI”) at the rate *χ*. Cases shed HI *V. cholerae* into the aquatic environment at a rate *ξ* and cases cease to be infectious at the rate *γ*. Non-HI vibrios shed into the aquatic environment lose viability at the rate δ_L_.

These ideas are expressed in terms of the following set of differential equations:



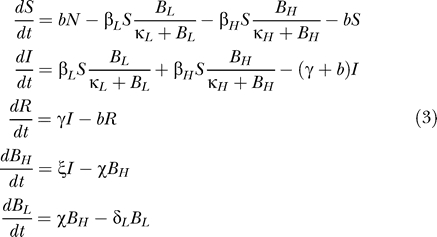



Representative values of the parameters appearing in these equations are listed in Table 1.

## Results

The expression for the basic reproductive number for this model is more complicated than in the simpler model and involves contributions from both the HI and non-HI states. By computing the dominant eigenvalue of the next-generation matrix corresponding to these equations, using the method described in Watmough and van den Driessche [12], it follows that



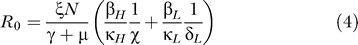



This is a new result. Biologically speaking, the first term in the parenthesis is associated with the number of new infections caused by HI vibrios, and the second term is associated with new infections caused by non-HI *V. cholerae*. As before, ξ/(γ + μ) is the average amount of *V. cholerae* shed per individual. The terms 1/χ and 1/δ_L_ are the expected times that vibrios remain in the HI and non-HI infectious states, respectively, before they decay into a non-infectious state (i.e., die in the environment or lose viability). Finally, β_H_ /κ_H_ and β_L_ /κ_L_ are the number of new cases generated in terms of the HI and non-HI vibrios, respectively, per unit time as measured by the number of ID_50_ concentrations.

An important question in cholera epidemiology is the relative importance of the HI and non-HI infectious routes. This quantity probably varies from place to place, depending on sanitation, population density, and hygiene standards. However, several relevant points can be made under a restricted set of assumptions.

First, we consider the case where contact with HI vibrios is about as frequent as contact with non-HI vibrios (β_L_ ~ β_H_). In such cases, the ratio of the HI to non-HI contributions to the *R_0_* is approximately (κ_L_δ_L_)/(κ_H_χ). Using parameter values cited in Table 1, this quantity is approximately 4.7, suggesting that the HI state is about five times more important than the non-HI state in generating cases early in the epidemic. As a thought experiment, consider a severe epidemic with both modes of transmission (*R_0_* ~ 18.2). If all the transmission from HI cholera were somehow prevented, but environmental (i.e., non-HI) transmission continued, the spread would be severely reduced (to *R_0_* ~ 3.2). In other words, the number of cases per case would be reduced by a factor of 5.7 at the beginning of the epidemic if there were no HI state.

We have used the model and the parameters in Table 1 to simulate the epidemic curves that might be observed in hypothetical community (*N* = 10,000 individuals; average lifespan = 1/*b* = 30 y) in the case that (a) no HI transmission exists versus (b) the case when a HI transmission occurs. We assume that a cholera epidemic begins when a single infectious individual enters a completely susceptible population and that the aquatic reservoir initially contains no *V. cholerae*. Figure 2 shows the number of cases as a function of time for the two scenarios. With no HI state (Figure 2A), cases peak after about 25 wk, at which time approximately 500 individuals are infectious, then decline until essentially none are present 50 wk following the introduction. With the HI state, (Figure 2B), cases peak in less than 2 wk, at which time approximately 3,500 people are infectious, then decline rapidly approximately 6 wk after the introduction. While, in reality, the depletion of susceptible population in the local neighborhood, control measures, and other changes in human behavior would slow the spread of cholera, the model qualitatively illustrates the role of the HI state in the timing and intensity of cholera outbreaks in naïve populations.

**Figure 2:**
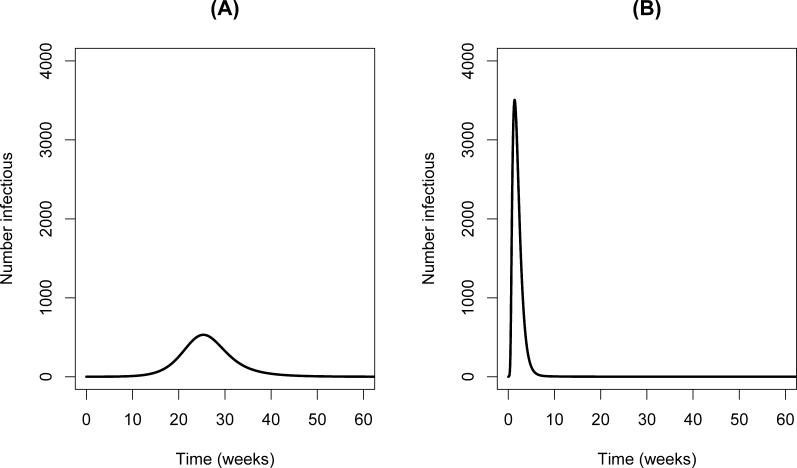
Number of Cholera Cases as a Function of Time (A) Model results when there is no HI state of *V. cholerae*. (B) Model results when there is a HI state of *V. cholerae*. Initial conditions for both (A) and (B): No *V. cholerae* present in the environment and all persons are susceptible except for a single active case present at the onset of the simulation.

Second, we make the stronger assumption that HI and non-HI cholera make equal contributions to *R_0_*; hence, in this scenario, there is less contact that occurs with HI cholera compared with non-HI cholera. Despite the reduction in the relative contact rates with HI cholera so that its contribution is equal to that of the non-HI, transmission through the HI route generates a greater share of the cases because it is able to complete several transmission cycles by the time one complete cycle is completed through the non-HI route. In other words, the generation time is substantially shorter when transmission occurs through direct transmission of HI cholera, and thus, cholera epidemics tend to be more explosive.

To illustrate, we set the contact rates so that the cases per case are equal (this occurs when β_H_ = β_L_ κ_H_ χ/(κ_L_δ_L_) and then plot the incidence (new infections per unit time) that occur from each transmission mode:



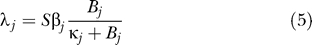



Incident cases due to the HI state correspond to *j = H* (*λ_H_;* broken trace in Figure 3A) and incident cases due to the non-HI state correspond to *j = L* (*λ_L_;* solid trace). Note that HI transmission produces the majority of new infections: in this case, the ratio of the height of the peaks is approximately 1.6. Moreover, the incidence due to the HI state peaks approximately 2 wk before the non-HI incidence peaks, and decays more rapidly than the incidence due to the non-HI state. The non-HI state is responsible for the slower dynamics of the outbreak while the HI state drives the fast dynamics. Another illustration of this point is depicted in Figure 3B, where we plot the relative contribution of each state to incident cases, λ_H_/(λ_H_ + λ_L_) (broken trace) and λ_L_/(λ_H_ + λ_L_) (solid trace) throughout the outbreak. From the beginning of the outbreak through approximately 5 wk, the HI state causes more cases than does the non-HI state. Thereafter, when most of the susceptible population has been infected, the non-HI state becomes more important.

**Figure 3:**
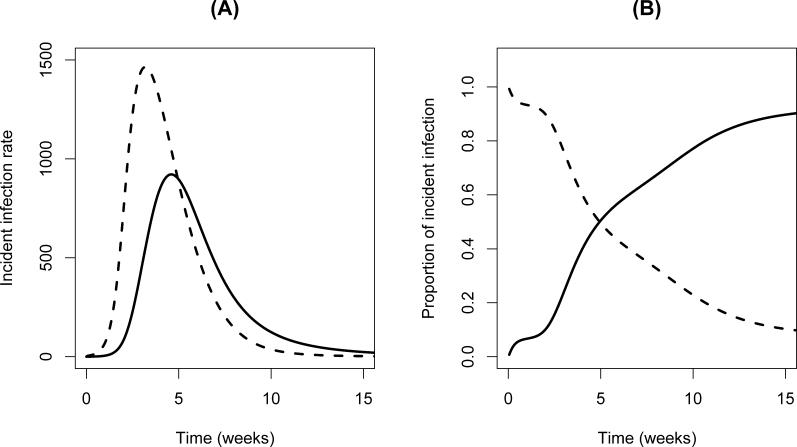
Roles Played by HI and Non-HI *V. cholerae* through the Course of an Epidemic when Each State Contributes Equally to *R_0_* Broken traces correspond to the HI state, and solid traces correspond to the non-HI state. Initial conditions are the same as in Figure 2B. (A) Rates at which incident infections appear. (B) Relative contribution of each state to incident cases.

The analysis above suggests that the HI state is extremely important in the transmission of epidemic cholera. How sensitive are these results to the particular model parameters used? Of all the parameters, β_H_ and β_L_ are most important for control and least well known. In order to illustrate the sensitivity of the model to the relative variation of these parameters, in Figure 4 we plot how *R_0_* depends upon the relative rates of contact with contaminated water, β_L_/β_H_, when the parameters of Table 1 are used. When β_L_ ~ β_H_, (contact with water contaminated with HI vibrios is as frequent as contact with water containing non-HI vibrios) *R_0_* is approximately 18.2, and as β_H_ becomes much smaller than β_L_ (contact with water contaminated with HI vibrios is much less frequent then contact with water containing non-HI vibrios), *R_0_* approaches 3.2, the value we expect when there is no contact with HI *V. cholerae*. When contact with water contaminated with HI vibrios is much more frequent then contact with water containing non-HI vibrios, *R_0_* will be very large (> 18.2).

**Figure 4:**
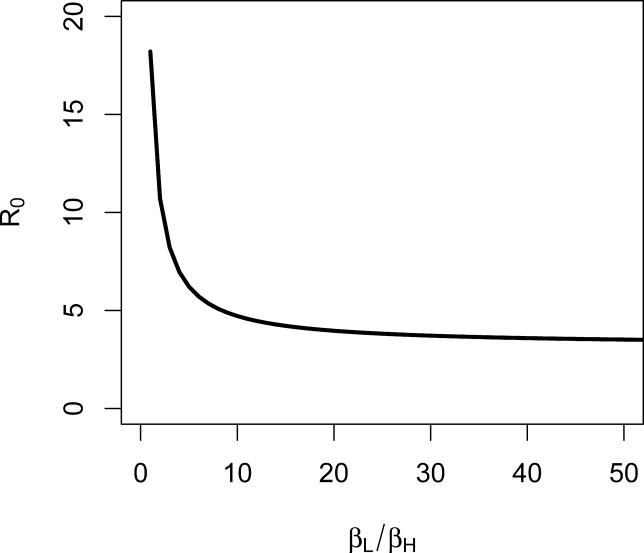
Sensitivity of the Basic Reproduction Ratio, *R_0_,* to the Relative Contact Rate with HI and Non-HI *V. cholerae* (β_L_/β_H_)

## Discussion

The epidemiological significance of the HI state is 2-fold. First, because HI vibrios are recently shed from individuals, they are close to humans, compared with vibrios in the environment. Consequently, HI vibrios are more likely to come into contact with other individuals (β_H_ > β_L_ in the model). The rapid decay of HI *V. cholerae* also implies that new infections will tend to be associated with recent infection of an infectious individual. Thus, even if HI vibrios are transmitted indirectly through local contamination (e.g., water or food), transmission will behave dynamically in populations as if it were direct. Second, the dramatic competitive advantage observed in [7] suggests the ID_50_ for HI *V. cholerae* is much lower than for non-HI *V. cholerae* (and thus κ_H_ << κ_L_ in the model). Even if the rates of exposure from quasi-direct transmission and indirect transmission through the environment are the same, for example, in the case of drinking well-mixed water from a contaminated supply such as a river polluted with raw sewage, *V. cholerae* in the HI state may be more epidemiologically significant because it requires a lower dose to cause disease and because the generation times are shorter. As demonstrated above, when *β_L_* ~ *β_H_*, for the parameters estimated from the literature (Table 1), *V. cholera* in the HI state contributes nearly six times more secondary cases than vibrios in the non-HI state at the beginning of the epidemic. Thus, we propose that these two effects combine to generate the explosive epidemic behavior historically observed in cholera outbreaks.

From a public health vantage point, these results suggest that careful attention should be given to differentiating the risk of exposure to “fresh” fecal contamination, versus “older” organisms acquired from environmental sources. Freshly excreted organisms, being many more times more infectious than less-recently excreted vibrios, are expected to dominate cholera transmission when proper hygiene is not practiced and effective waste disposal is not available. Likewise, older organisms are expected to dominate cholera transmission dynamics when good hygiene and effective waste engineering practices are followed. Real epidemics may combine elements of both—isolated cases from environmental cholera followed by clusters of cases due to local transmission. Such results re-emphasize the importance of focusing prevention efforts on improving hygiene, including frequent hand-washing, sewage disposal, good water, and food-handling practices in the home and in food service establishments (restaurants, street vendors) where contact with freshly shed vibrios is most likely.

Interestingly, these results suggest a possible explanation for the puzzle of why cholera was controlled in Europe and the United States before the widespread disinfection of water. In the mid- to late-1800s, sewage disposal systems began to be engineered and built in municipal areas. Because these systems dramatically increased hygiene standards and decreased the opportunity for contact with fresh feces (resulting in a smaller *β_H_*), the HI state of the pathogen played a less important a role in transmission. Of course, if such systems transported sewage to locations where non-HI vibrios could contaminate drinking sources, transmission remained possible, albeit at a greatly reduced rate. We postulate that the HI state would have decayed during transport, so that disease due to contaminated water supply would have been associated with non-HI *V. cholerae*. In fact, in the US decades passed before cholera was effectively wiped out following introduction in the early 1830s [13]. This provides an important implication for developing regions vulnerable to cholera outbreaks: even if water purification is not possible, there are still public health benefits to improved hygiene and sewage management systems.

The observation of the HI state was made experimentally for *V. cholerae* O1 Inaba El Tor [7]. To our knowledge, no observations under field conditions have appeared in the literature. Additional research is therefore needed to further characterize such states. The recent description of hyperinfectious *V. cholerae* O1 El Tor in a mouse model should aid in such research [8]. Our mathematical model demonstrates the potential importance of such a transient state for epidemic transmission, and highlights the need to search for analogous states in related species of *V. cholerae*. Moreover, if transient states of elevated infectivity exist in other pathogens besides *V. cholerae,* we anticipate they will be if key importance to epidemic transmission.

**Patient Summary**

**Background**

Cholera remains a public health problem in countries without access to safe drinking water and adequate sanitation. People can get cholera from contaminated food or water, or from contact with feces or vomit of patients. Researchers are trying to develop theoretical models for infectious diseases, including cholera, that allow them to understand how an outbreak happens, how it could best be contained, and how it might be prevented. Existing models have not been able to explain actual cholera outbreaks very well.

**Why Was This Study Done?**

Three years ago, another group of researchers reported that cholera bacteria isolated from the stools of sick patients were much more infectious than those found in contaminated water. (They compared the two by exposing mice to a mix and determining which bacteria made the mice sick.) Those researchers proposed that the infection of a human patient (i.e., the exposure to an environment that is quite different from their regular freshwater ponds) changes the cholera bacteria. As a result, for a short period of time, the bacteria become more infectious. The researchers who did the present work are interested in cholera modeling. They wanted to incorporate the idea of a short-lived hyperinfectious state and see whether the resulting model might better explain cholera outbreaks.

**What Did the Researchers Do and Find?**

They assumed that the number of hyperinfectious bacteria (i.e., those from fresh feces of a sick patient) necessary to cause disease was smaller than the number from contaminated food or water and that this period of hyperinfectivity was very short. They adjusted a model to take this into account and found that it could now better explain the explosive nature of cholera outbreaks.

**What Does This Mean?**

The original results suggesting that passage through humans makes cholera bacteria more infectious were based on experiments in mice. That a cholera model fits reality better when it assumes the existence of such a hyperinfectious state makes it likely that the findings are relevant to disease transmission in humans. The findings also have public health implications: they emphasize the need to avoid contamination with fresh patient feces in the context of overall improved hygiene.

**Where Can I Find More Information Online?**

The following Web sites provide information about cholera.

Wikipedia pages on cholera:

http://en.wikipedia.org/wiki/Cholera

WHO pages on cholera:

http://www.who.int/topics/cholera/en/

Cholera pages from the U.S. Centers for Disease Control and Prevention:

http://www.cdc.gov/ncidod/dbmd/diseaseinfo/cholera_g.htm

## Abbreviation

HI: hyperinfectious
